# A review of demodulation techniques for multifrequency atomic force microscopy

**DOI:** 10.3762/bjnano.11.8

**Published:** 2020-01-07

**Authors:** David M Harcombe, Michael G Ruppert, Andrew J Fleming

**Affiliations:** 1 School of Electrical Engineering and Computing, The University of Newcastle, Callaghan, NSW, 2308, Australia

**Keywords:** atomic force microscopy (AFM), multifrequency, demodulation, Kalman filter, Lyapunov filter, digital signal processing, field-programmable gate array (FPGA)

## Abstract

This article compares the performance of traditional and recently proposed demodulators for multifrequency atomic force microscopy. The compared methods include the lock-in amplifier, coherent demodulator, Kalman filter, Lyapunov filter, and direct-design demodulator. Each method is implemented on a field-programmable gate array (FPGA) with a sampling rate of 1.5 MHz. The metrics for comparison include the sensitivity to other frequency components and the magnitude of demodulation artifacts for a range of demodulator bandwidths. Performance differences are demonstrated through higher harmonic atomic force microscopy imaging.

## Introduction

Atomic force microscopy (AFM) [1] has enabled innovation in nanoscale engineering since it was invented in 1986 by Binnig and co-workers. Atomic-scale topographical resolution is achieved by sensing the interaction between a sharp microcantilever probe and the sample [2]. Initial operation was in constant-force contact-mode, where a static deflection is maintained through a constant contact force [3].

In dynamic imaging modes [4], the cantilever is driven at, or near, a resonance frequency, which establishes the requirement for demodulation in AFM. In intermittent-contact constant-amplitude AFM [5], a constant cantilever oscillation amplitude is maintained by feeding back the demodulated fundamental amplitude of the deflection signal. The imaging of delicate biological samples [6–8] is particularly suited to intermittent-contact AFM [9] when tip–sample contact is gentle.

Environmental damping has a large effect on the quality factor (*Q*) of the cantilever. Values can range from as low as *Q* ≈ 1 in liquid [10], up to *Q* ≈ 10,000 in ultra-high vacuum [11]. This affects the mechanical bandwidth of the cantilever according to the expression *f*_−3dB_ = *f*_0_/2*Q*, where *f*_0_ is the fundamental resonance frequency. Assuming all other components in the *z*-axis feedback loop are also working at high speed [3], a low quality factor can demand a fast demodulator [12].

Multifrequency AFM (MF-AFM) is a major field within dynamic mode AFM. It involves studying multiple frequency components in the cantilever oscillation during tip–sample interactions [13]. Observing higher eigenmodes of the cantilever [14], higher harmonics of the fundamental resonance [15] and intermodulation products [16] have been shown to provide further nanomechanical sample information. These include properties such as sample elasticity, stiffness and adhesiveness [17], which are mapped simultaneously with the topography. Acquiring these observables requires the accurate demodulation of amplitude and phase of multiple frequency components.

Small interaction forces associated with higher-harmonic AFM have been imaged in free air [18] as well as liquid [19]. This has lead to relatively large biological objects being imaged including viruses [20] and cells [21]. Multimodal AFM, where two or more resonance frequencies are driven, has theoretical foundations for determining secondary sample properties such as Young’s modulus [13,22]. Applications include the imaging of secondary properties of proteins [23] and polymers [24]. Intermodulation AFM actively drives the cantilever slightly below and above resonance with a two-tone drive. Compared to higher-harmonic AFM, this technique has more enhanced non-linear interactions [25]. Intermodulation products present in the cantilevers motion have been shown to be sensitive to material and chemical contrast [16,26], leading to enhanced nanomechanical insights [27]. Regardless of which MF-AFM technique is performed, the demodulator is an essential component for acquiring observables to characterize the sample.

Previously, the authors conducted an in-depth comparison of conventional and novel demodulation techniques for single-frequency amplitude-modulation atomic force microscopy [28]. It was found that conventional high-speed non-synchronous demodulators are incompatible with MF-AFM, due to the lack of robustness against unwanted frequency components [28]. These include the peak-hold [12], peak detector [29] and RMS-to-DC [30] conversion demodulators. In contrast, synchronous demodulators have been shown to provide accurate estimates in the presence of other frequency components [28]. As a result, MF-AFM experiments usually employ multiple lock-in amplifiers in parallel. However, this introduces an inherent bandwidth limitation as high-frequency mixing products must be low-pass filtered [28,31].

Motivated by improving high-speed MF-AFM demodulation capabilities, a multifrequency Kalman filter was developed [32]. It outperformed a commercially available lock-in amplifier in terms of both tracking bandwidth and noise performance. However, a major disadvantage of the Kalman filter is its implementation complexity. This heavily limits the achievable sampling rate and ability to track a large number of signals. To alleviate this issue, the Lyapunov filter [33] was established, which is computationally more efficient than the Kalman filter while achieving similar performance [34]. This was extended to a multifrequency Lyapunov filter, which has seen success in higher-harmonic AFM for both amplitude and phase-contrast imaging [35–36]. A limitation, common to both the Kalman and the Lyapunov filter, is a fixed 1st-order response, which has motivated the development of techniques for the direct design of the demodulator frequency response [37–38].

This article aims to provide a rigorous experimental comparison of MF-AFM demodulation techniques. This includes the conventional lock-in amplifier and coherent demodulator, as well as the recently proposed Kalman filter, Lyapunov filter and direct-design method. For a fair comparison, each system is implemented on the same FPGA platform with a common sample rate. The sensitivity to unwanted frequency components for both low and high bandwidths is assessed along with implementation complexity. A final experimental comparison is conducted through higher-harmonic AFM imaging for both low and high tracking bandwidths.

## Multifrequency AFM Modulation and Demodulation Fundamentals

### Cantilever deflection signal model

A single component of the cantilever deflection signal is modeled as a sine wave with carrier frequency *f_i_*, time-varying amplitude *A_i_*(*t*) and phase ϕ*_i_*(*t*), that is of the form

[1]
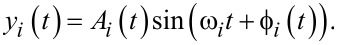


For a better readability, explicit time-dependencies on the amplitude *A*(*t*) and phase ϕ(*t*) are dropped from this point onward. By extension, a deflection signal consisting of multiple frequencies is given by

[2]
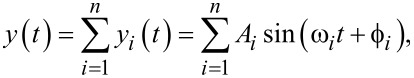


where *i* = 1, 2, …, *n* denotes the *i*-th modeled frequency. An alternative representation of a single signal component is of the linearly parameterized form

[3]
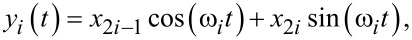


where *x_2i−1_* and *x_2i_* represent quadrature and in-phase components respectively. This is convenient for MF-AFM, as the time-varying amplitude and phase of each frequency can be recovered by the output equations

[4]
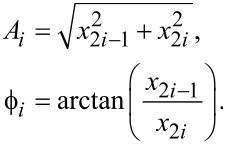


### Modulation

**Figure 1:**
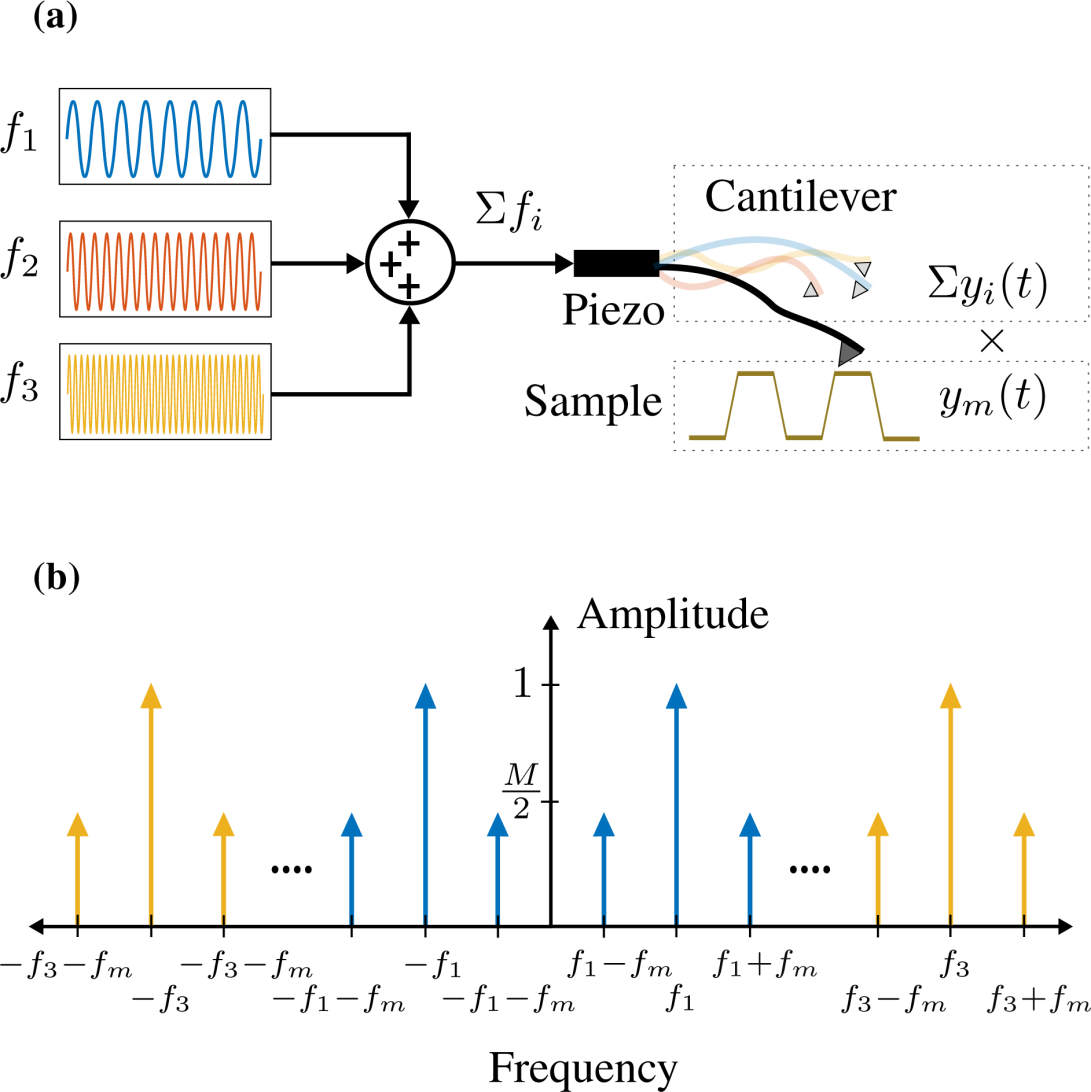
(a) Schematic diagram of sample topography amplitude-modulating a cantilever the oscillation of which consists of multiple frequencies. (b) Double-sided amplitude frequency spectrum of a cantilever oscillating at multiple frequencies Σ*y_i_*(*t*) while being amplitude-modulated by the sample topography *y*_m_(*t*).

An amplitude-modulated signal (double-sideband full carrier) is obtained by mixing a modulating signal *y*_m_(*t*) at a frequency ω_m_ = 2π*f*_m_ with a carrier signal *y_i_*(*t*). The modulating signal oscillates at a frequency that is significantly slower than the carrier frequency ω*_i_*. Figure 1a illustrates a cantilever driven at multiple frequencies being amplitude-modulated by a sample topography. In MF-AFM, the cantilever deflection signal contains frequency components originating from the fundamental resonance mode, as well as from higher eigenmodes and/or harmonics. If for simplicity we assume unity amplitudes, then amplitude-modulation of a distinct frequency component at ω*_i_* is described by

[5]
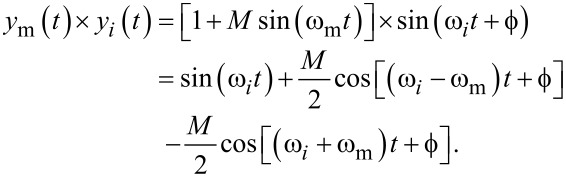


Here, *M* is the modulation index, which for AM signals is the ratio of the peak value of the modulated signal relative to the carrier. Equation 5 shows that the modulation process creates distinct frequencies components at *f_i_* and *f_i_* ± *f*_m_. The latter components are termed the upper and lower sidebands and are centered symmetrically around the carrier frequency as illustrated in Figure 1b. As the modulating frequency increases, the sidebands move away from the carrier up until the limit where the left sideband is at DC and the right sideband is at 2*f_i_*. The scenario where *f*_m_ *> f_i_* is not of practical interest, as the amplitude changes would need to be faster than the cantilever oscillation frequency.

### Demodulation

Demodulation is the process of estimating the modulating signal (sample) associated with a carrier frequency. Demodulators can be classified as either synchronous or non-synchronous. Non-synchronous methods do not require a reference oscillator. However, these methods are incompatible with MF-AFM, due to their inability to reject unwanted frequency components [28]. For this reason, these techniques are not discussed in this article. Synchronous demodulation techniques employ a reference oscillator and can be categorized as either open-loop or closed-loop, depending on whether they use feedback to estimate parameters. Open-loop demodulators include the lock-in amplifier and coherent demodulator, while closed-loop methods include the Kalman filter, Lyapunov filter, and direct-design demodulator.

### Performance metrics

In a previous work [28], the performance of single-frequency AFM demodulators was assessed by measuring the magnitude of demodulation artifacts and the sensitivity to measurement noise. However, multifrequency AFM applications require an additional metric due to the large number of potentially closely spaced frequencies. For example, higher-harmonic imaging with single-frequency excitation results in small harmonic amplitudes that must be estimated in the presence of both noise and much larger fundamental and/or harmonic components [19,36]. The performance of the demodulator in this regard can be quantified by a metric herein referred to as the off-mode rejection (OMR).

**Figure 2:**
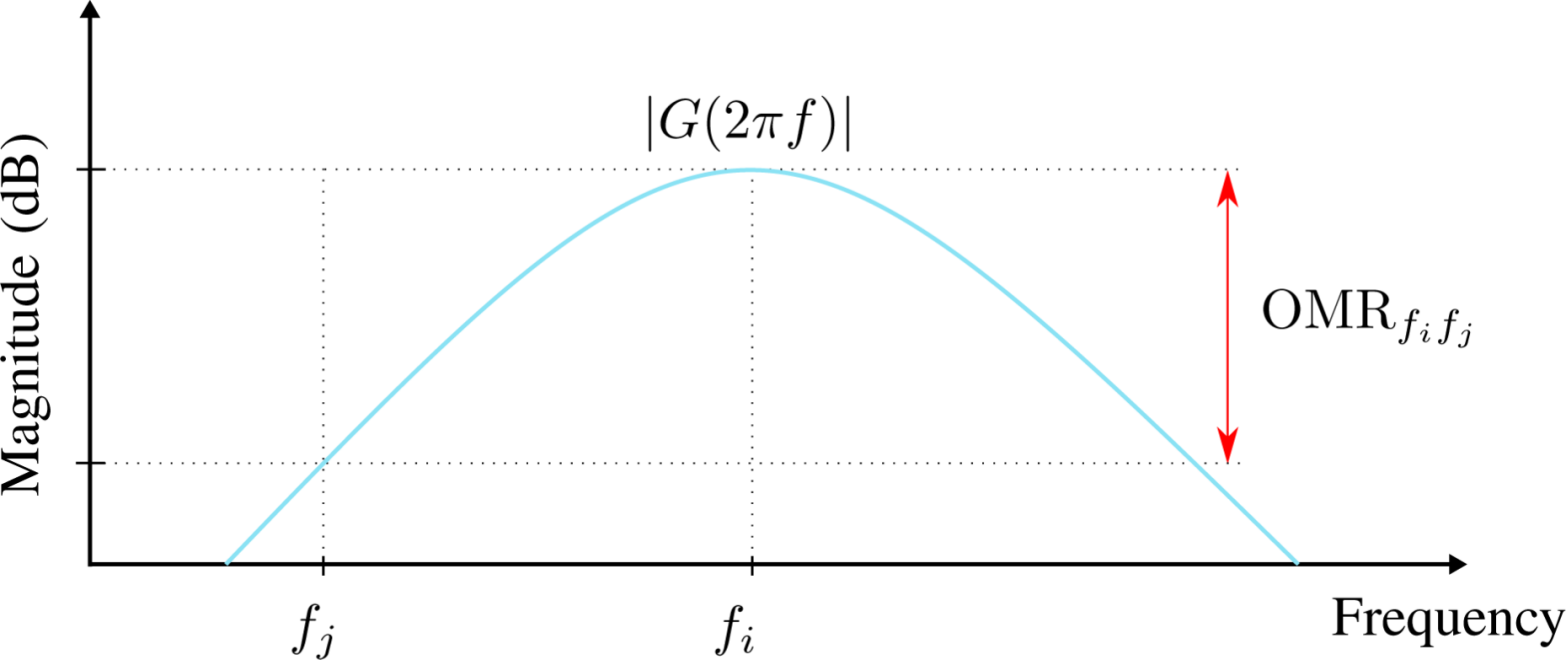
Visualization of off-mode rejection in the frequency domain for a demodulator magnitude frequency response |*G*(2π*f*)| at *f_i_* with respect to *f_j_*.

OMR is defined as the gain ratio between a modeled carrier frequency *f_i_* and another frequency *f_j_* as visualized in Figure 2. It can be evaluated by

[6]
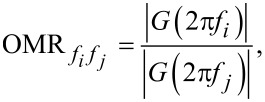


where *G*(2π*f*) is the demodulator frequency response. Additionally, implementation complexity is qualitatively discussed. It is assessed according to the maximum achievable sampling rate, timing requirements and computational scalability when modeling additional channels.

## Review of Multifrequency Demodulation Methods

### Lock-in amplifier

The multifrequency lock-in amplifier (LIA) [28,39–41] operates by multiplying an input signal described by Equation 2 with parallel in-phase and quadrature sinusoids tuned to frequencies the amplitude and phase of which are of interest. For simplicity, consider an ideal input signal consisting of a single sinusoid with a frequency ω*_i_*, applied to a lock-in amplifier tuned to ω*_i_*. During the mixing process, the following intermediate signals are generated

[7]
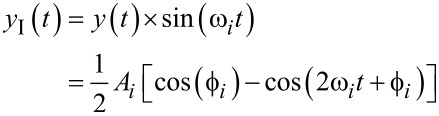


and

[8]
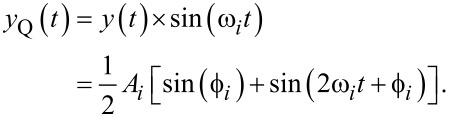


From Equation 7 and Equation 8 it can be seen that in addition to the desired amplitude and phase, mixing products are generated at twice the carrier frequency 2ω*_i_*. If the input contains more than one sinusoid and/or a noise process, further undesired frequency components are present in the intermediate signals. These high-frequency mixing components and noise terms are removed by employing a low-pass filter, the cut-off frequency of which is determined by making a trade-off between tracking bandwidth and 2ω*_i_* ripple suppression [28]. Additionally, lock-in amplifiers should always be AC-coupled as any residual DC offset in the input signal (Equation 2) will generate a mixing component at ω*_i_*.

**Figure 3:**
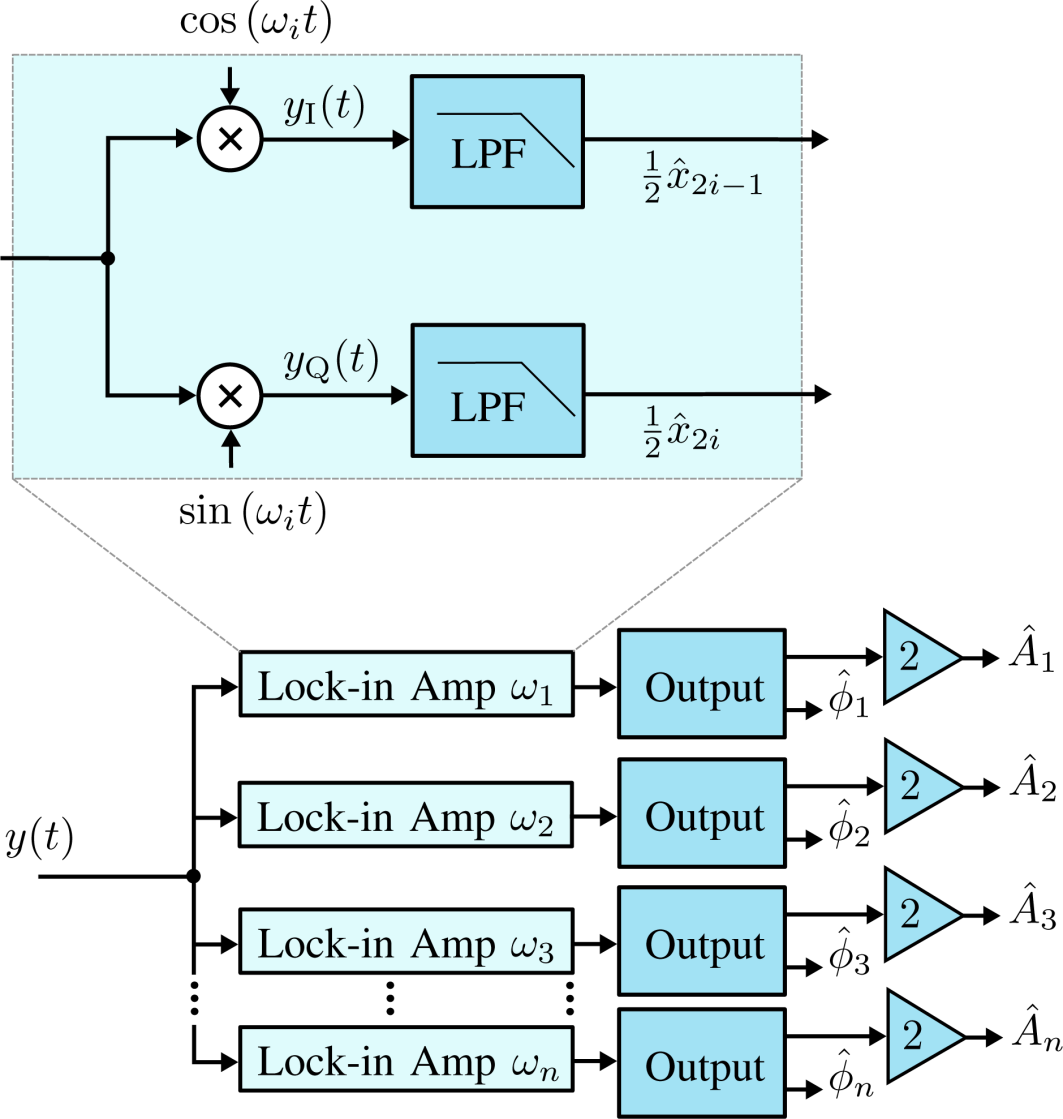
Functional block diagram of the multifrequency lock-in amplifier implementation. The zoom-box displays the functional block diagram of a single lock-in amplifier.

The functional block diagram of the multifrequency lock-in amplifier is shown in Figure 3. Here, it can be seen that multiple frequencies are tracked by running several lock-in amplifiers in parallel, with each oscillator tuned to a specific frequency ω*_i_*. The required components for digital implementation of each lock-in amplifier are a direct digital synthesizer (DDS) to generate the sine and cosine mixing signals, two multipliers, two low-pass filters and an output block. The output block, which calculates amplitude and phase, is described by Equation 4, meaning the square-root and arctan functions are required. Typically the phase is calculated by using either a polynomial approximation [42] or the CORDIC algorithm [43].

### Coherent demodulator

The multifrequency coherent demodulator is a digital demodulation method based on mixing and precise integration over a fixed time window [28,44–47]. Conceptually, it is a digital lock-in amplifier that utilizes mixing with in-phase and quadrature sinusoids

[9]
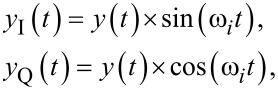


and implements low-pass filtering of mixing products and any other additional unwanted frequency components through precise fixed-length numerical integration [45]. If the input signal is a pure sinusoid (Equation 1) and the integration period *T* is chosen to be an integer multiple of the drive signal period, *T* = *mT_i_*, the integrals over *y*_I_(*t*) and *y*_Q_(*t*) evaluate exactly to the in-phase and quadrature states

[10]
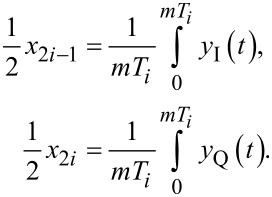


**Figure 4:**
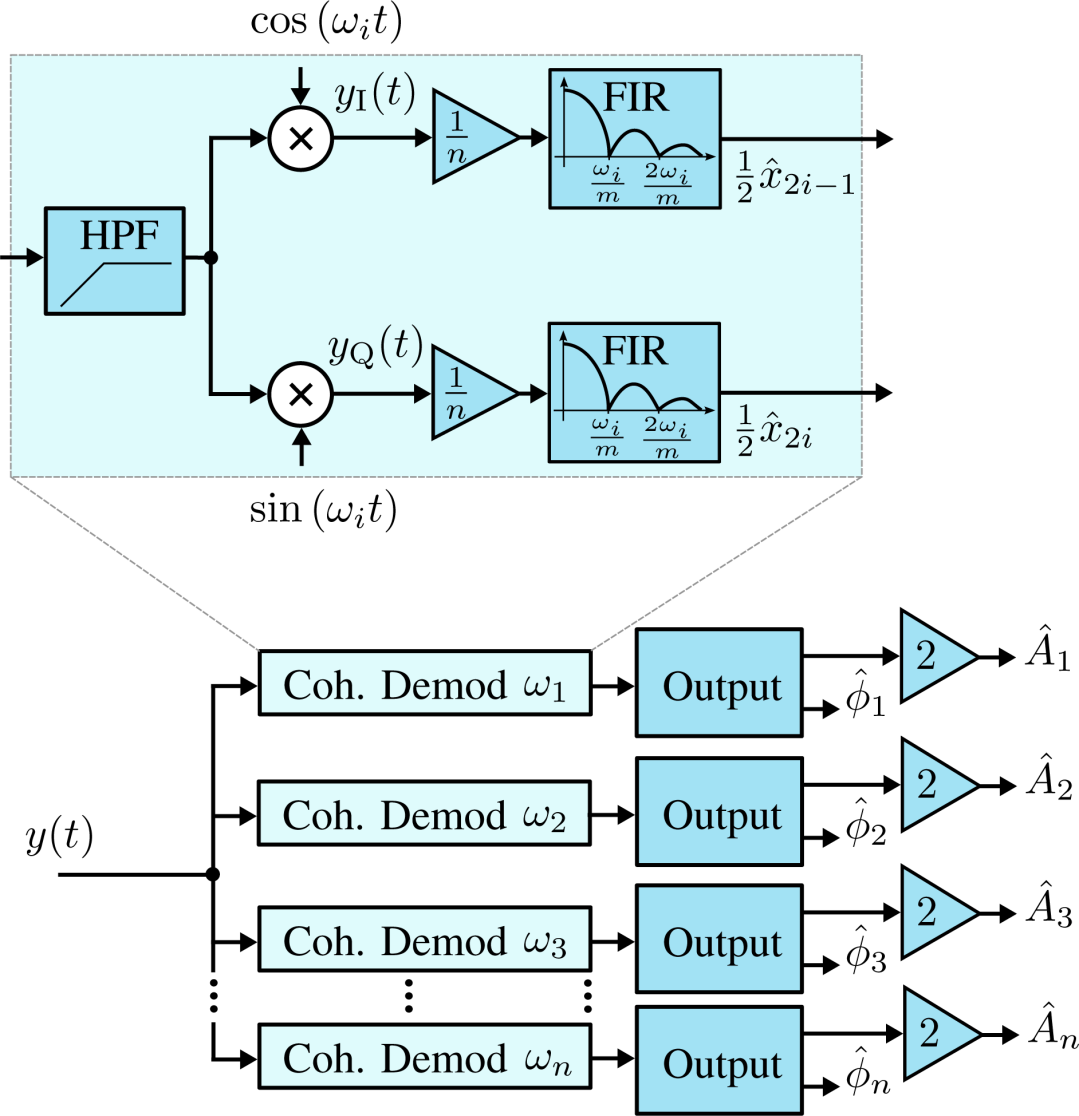
Functional block diagram of the multifrequency coherent demodulator implementation. The zoom-box displays the functional block diagram of a single coherent demodulator.

The functional block diagram of the multifrequency coherent demodulator is shown in Figure 4. It requires the same components as the lock-in amplifier, although the method in which the low-pass filter is implemented is different. Advanced implementation details can be found in the literature [28,45]. Practitioners should pay strong attention to timing considerations, otherwise the desired low-pass filtering effect will not occur.

For Equation 10 to hold, the integration period must be an integer multiple of the sampling period, *nT*_s_ = *mT_i_*, where *n* is the number of samples in the integration. Since an arbitrary sample-to-carrier frequency ratio *F*_s_/*f_i_* is rarely an integer, this condition is hard to meet. Therefore, a practical solution is to find the smallest *n* such that *nT*_s_ ≤ *mT_i_* ≤ (*n* + 1)*T*_s_ and perform a partial integration over the last sampling interval [45]. Such precise control over the integration period is achievable in digital systems, although the implementation of this method is still challenging.

The discrete-time integration in Equation 10 yields a very useful finite impulse response (FIR) filter, the frequency response of which is a sinc(·) function with zeros occurring at integer multiples of the oscillation frequency [28]. Unlike the lock-in amplifier, this allows the coherent demodulator to achieve low-noise output estimates at high tracking bandwidths since it strongly rejects 2*f_i_* mixing products [28]. In addition, this zeroing characteristic can provide strong attenuation of unwanted harmonics and intermodulation products. This has lead to the multifrequency coherent demodulator being successfully applied to intermodulation AFM [26–27].

### Kalman filter

The Kalman filter [48] has seen practical application in many fields including inertial navigation [49], robotics [50], and economics [51]. The Kalman filter uses a recursive algorithm to minimize the error between modeled and measured information to estimate an unknown process variable. Specifically, if the modeling and measurement noise processes have a Gaussian distribution, the Kalman filter produces an optimal estimate of a variable in the least-squares sense by minimizing the variance [52]. Fundamental to its operating principle, the Kalman filter utilizes a linear model of system dynamics and feedback of the state variables to update the Kalman gains, which controls the tracking bandwidth.

When the time-varying system is discretized for *t* = *kT*_s_, where *T*_s_ is the sampling period, the process model of the Kalman filter is established as

[11]
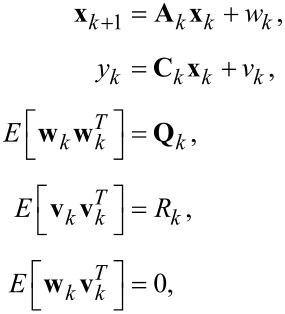


where *w_k_* and *v_k_* are the process noise and measurement noise with respective covariance matrices **Q***_k_* and *R_k_*. The state and output matrix are described by

[12]
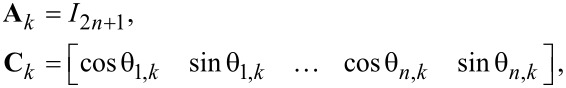


where *I_2n+1_* is the identity matrix of dimension 2*n* + 1, *n* is the number of modeled frequencies, θ*_i,k_* = ω*_i_kT*_s_. In this representation, quadrature *x_2i−1,k_* and in-phase *x_2i,k_* states are assumed to be random variables describing the states of Equation 3.

The parameters **Q***_k_* and *R_k_* dictate the amount of uncertainty in the model and the measurement noise, respectively. To simplify tuning of the filter during operation it is recommended to fix *R_k_* such that it reflects the standard deviation σ of the Gaussian noise in the input signal from the sensor *y*(*t*) (*R* = σ^2^). This leaves **Q***_k_* as the only tuning variable that directly influences the Kalman gains and sets the tracking bandwidth.

**Figure 5:**
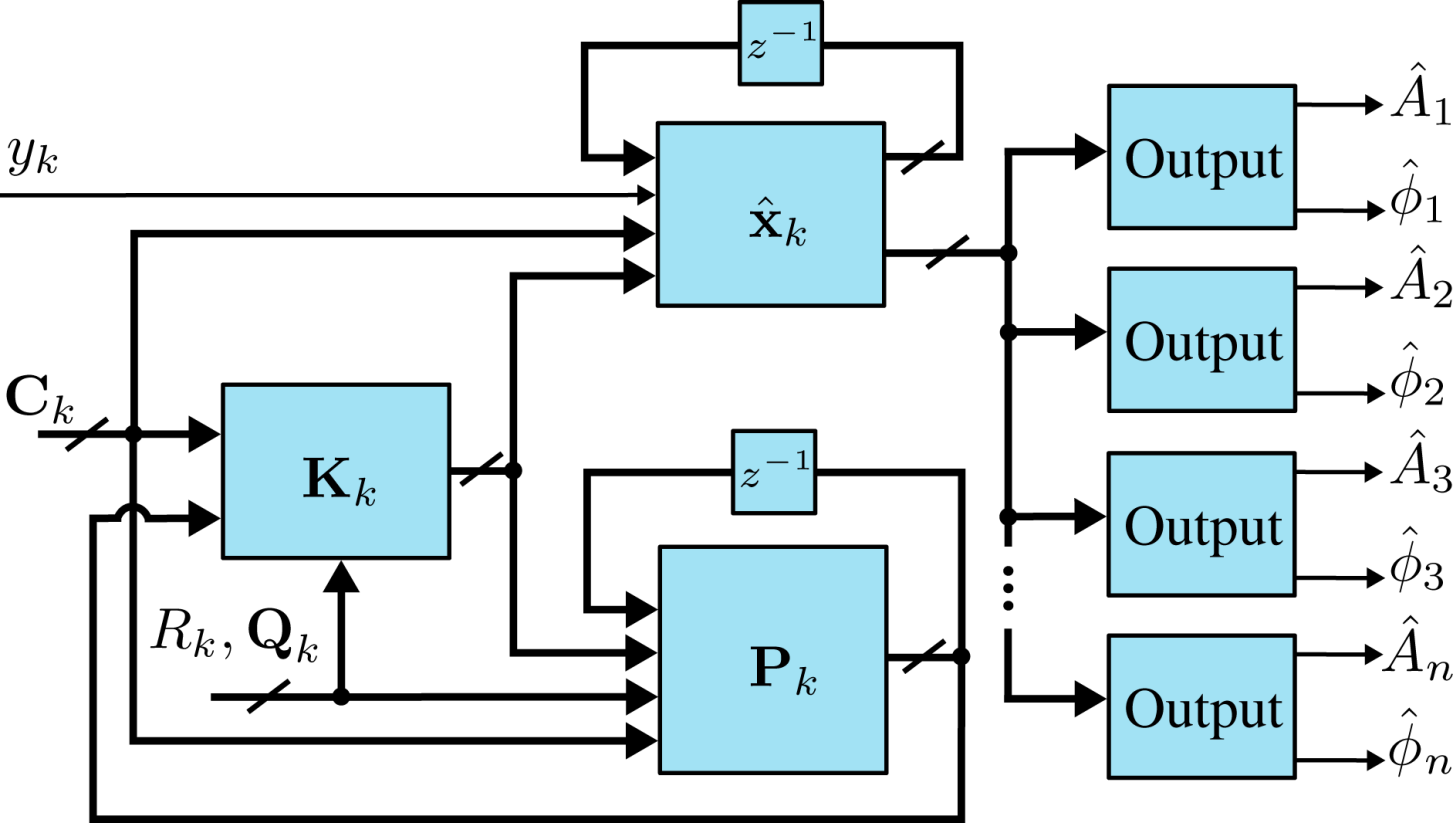
Functional block diagram of the Kalman filter implementation.

The functional block diagram of the Kalman filter implementation is shown in Figure 5, it follows the standard recursive algorithm equations [53–54]. The prediction step is computed as

[13]
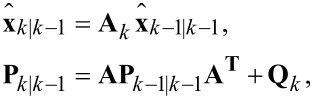


where **P***_k|k−1_* denotes the predicted covariance matrix. This is followed by the Kalman gain and state measurement updates

[14]
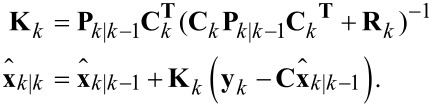


Lastly, the covariance matrix is updated according to

[15]
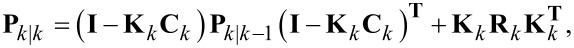


which is in Joseph form, i.e., it is naturally symmetric and positive definite. These properties can be exploited in the implementation to reduce memory and computation requirements. In addition, it is the most numerically stable form of the covariance matrix and remains convergent and non-deterministic for any selection of **Q***_k_* and *R_k_*[53].

Specific amplitude and phase of a modeled frequency ω*_i_* are shown to be recovered by employing the output equations in Equation 4. Although this method is simple to tune in real-time, a disadvantage is the fixed 1st-order response. Also, the Kalman filter equations have a complexity of 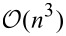 for *n* modeled frequencies resulting in significant computational requirements beyond three modeled frequencies. This system representation has seen success in tracking power system voltage phasors [55] and more recently high-speed AFM [31–32].

### Lyapunov filter

The Lyapunov filter [33,35–36] also uses a model-based feedback approach to obtain amplitude and phase of signals at desired frequencies. Under certain conditions, the Lyapunov filter has been shown to be equivalent to the Kalman filter [33]. However, the Lyapunov filter uses a tunable scalar gain γ instead of updating covariance matrix and Kalman gain equations. This gives the Lyapunov filter a computational complexity of 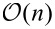 as additional frequencies are modeled, a significant improvement over the Kalman filter.

**Figure 6:**
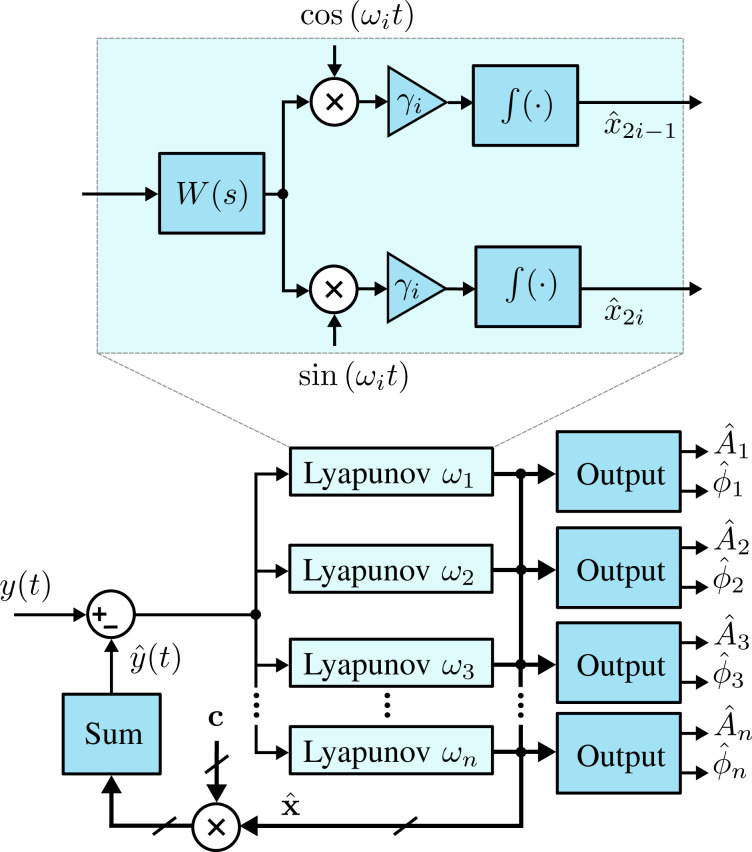
Functional block diagram of the multifrequency Lyapunov filter implementation. The zoom-box displays the functional block diagram of a single Lyapunov filter.

A key property of the Lyapunov filter is exponential convergence of the estimated states [56], with the tunable loop gain γ governing the speed of convergence. The multifrequency Lyapunov filter is implemented as parallel linear observers tuned to a particular frequency ω*_i_*, as depicted in Figure 6. An error signal is generated by feeding back an estimate of the input signal as per Equation 2, obtained from the parameterized states of each individual filter. Regulation of this error through feedback leads to the much desired suppression of the high-frequency mixing components.

The update law for the Lyapunov filter [33,36] for multiple frequencies is written as

[16]
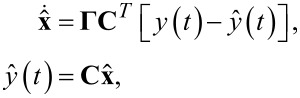


where

[17]
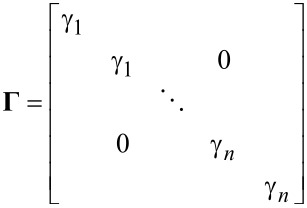


and

[18] $$C=\left[\cos\left(\omega_{1}t\right)\;\sin\left(\omega_{1}t\right)\ldots \cos\left(\omega_{n}t\right)\;\sin\left(\omega_{n}t\right)\right].$$

In this form, 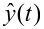 represents the estimated input signal and the amplitude *A_i_* and phase ϕ*_i_* estimates are found by applying Equation 4 to each quadrature and in-phase pair of 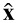. A key property to ensure exponential convergence of 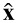 to **x** is to guarantee that **C** is persistently excited [56]. Convergence is shown for the single-frequency filter in [33] and can easily be extended for the multifrequency case. Furthermore, exponential convergence of 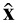 means that 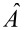 and 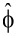 also converge. This system representation has been shown to perform similarly to the Kalman filter [28], which is advantageous given its implementation simplicity. Recently, it has been used for higher-harmonic AFM for both amplitude and phase-contrast imaging [35–36].

### Direct-design method

The direct-design method [37] also utilizes model-based feedback to obtain the amplitude and phase of signals at desired frequencies. However, intrinsic to its design methodology is the ability to implement an arbitrary filter response with a specified filter order and linearity in the bandpass region. For example, a demodulator can be implemented the frequency response of which resembles a Butterworth or Chebyshev filter with a desired filter bandwidth and order. This alleviates the limited 1st-order response of the Kalman and Lyapunov filters, creating stronger rejection of unwanted frequency components. This occurs while maintaining benefits such as low noise and low computational complexity.

**Figure 7:**
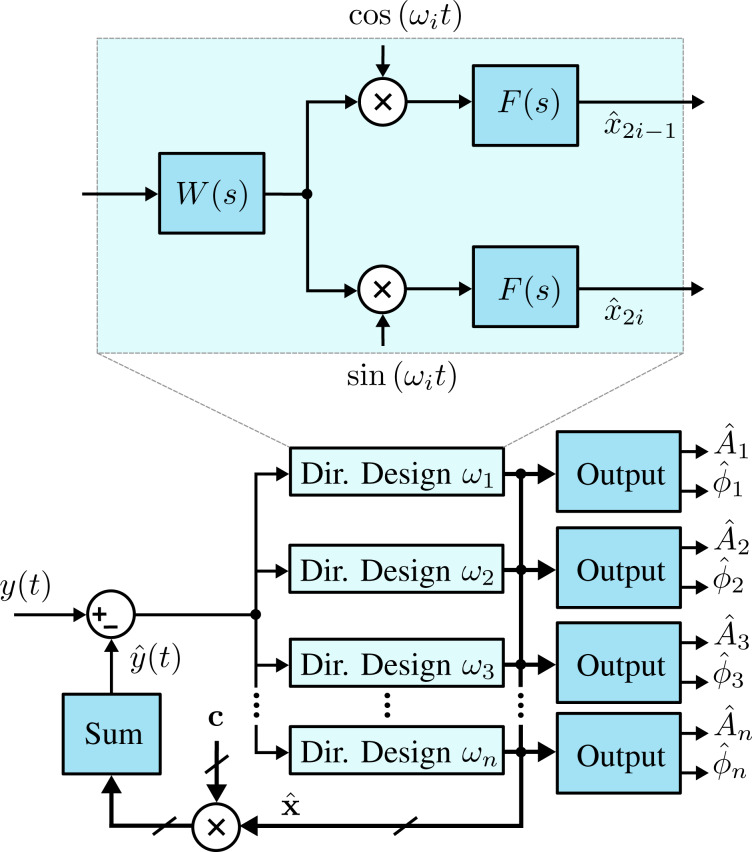
Functional block diagram of the multifrequency direct-design filter implementation. The zoom-box displays the functional block diagram of a single direct-design filter.

In order to obtain an arbitrary demodulator response, consider the functional block diagram in Figure 7, where the integrator of the Lyapunov filter is replaced by the transfer function *F*(*s*). In this form, the direct-design demodulator follows a modulated–demodulated control loop [57] with a unity plant. This method differs from the Lyapunov filter as it does not set the pre-filter to *W*(*s*) = 1, instead it utilizes *W*(*s*) as part of the design of a desired closed-loop response. In the original work [37] on the direct-design method, a useful and relatively simple design methodology is detailed. Firstly, *F*(*s*) is set as

[19]
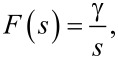


meaning the closed-loop equivalent transfer function is

[20]
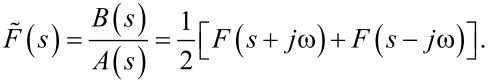


The pre-filter *W*(*s*) = *P*(*s*)/*L*(*s*) is then found according to the pole-assignment equation

[21]
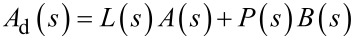


to achieve a specified closed-loop response.

For example, to implement a 2nd-order bandpass Butterworth prototype, the closed-loop transfer function is

[22]
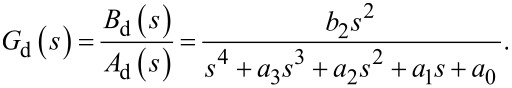


Here, *b*_2_ and *A_i_* are the filter coefficients the values of which are calculated based on the chosen filter order and bandwidth around the modeled frequency ω*_i_*. As the desired closed-loop polynomial has five coefficients, the coefficients of the pre-filter *W*(*s*) are of the form

[23]
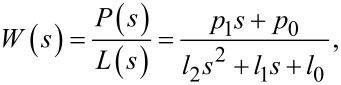


and are able to be obtained by solving Equation 21.

The existing literature on direct-design demodulation techniques [37–38] is concerned with single-frequency applications. However, this article demonstrates the performance advantages that can also be achieved in multifrequency applications.

### Summary

Table 1 compares the multifrequency demodulation techniques discussed in this section. Two distinct categories of synchronous demodulators can be seen; those that employ low-pass filtering of mixing products in open-loop configurations and those that use closed-loop model-based feedback to regulate the error. As shown in a previous work [28], the closed-loop methods are able to maintain very high tracking bandwidths, achieving single-cycle convergence (*f*_−3dB_ ≈ *f*_i_) with optimal noise performance.

**Table 1:** Summary of multifrequency demodulation estimation methods.

method	tuning	configuration	order	references

lock-in amplifier	low-pass filter	open-loop	specified *n*	[28,39–41]
coherent demodulator	number of hold cycles	open-loop	specified *n*	[28,44–47,58]
Kalman filter	**Q***_k_*	closed-loop	1st	[28,31–32,48,53]
Lyapunov filter	**Γ**	closed-loop	1st	[28,33,35–36,59]
direct-design method	desired poles	closed-loop	specified *n*	[37–38]

## Results and Discussion

### Experimental setup

The multifrequency demodulation techniques detailed in the previous section were implemented on a Xilinx Kintex-7 KC705 evaluation board (model: XC7K325T) paired with a DC-coupled high-speed 4DSP input/output (I/O) card (model: FMC151). The FPGA clock is synchronized with the high-speed I/O card at 240 MHz. The I/O card has a two-channel 14-bit analog-to-digital converter (ADC) and a two-channel 16-bit digital-to-analog converter (DAC), which sample at 250 MHz and 800 MHz, respectively. All demodulation methods were run at a nominal sampling frequency of *F*_s_ = 1.5 MHz.

### Implementation

Because of the high complexity, the sampling rate off the Kalman filter implementation was set to *F*_s_ = 1.5 MHz, which was the maximum achievable for three modeled frequencies employing floating point precision to ensure covariance matrix stability and a computationally optimized implementation [32].

The Lyapunov filter and direct-design method achieve sampling rates of *F*_s_ = 7 MHz for three modeled frequencies. This is due to the reduced complexity compared to the Kalman filter, floating point precision was also used to implement these methods.

The open-loop methods include the lock-in amplifier and coherent demodulator, which are able to achieve *F*_s_ = 120 MHz for three modeled frequencies. In contrast to the closed-loop methods, the open-loop methods are compatible with pipelined fixed-point implementation, which results in significantly increased maximum sampling rates and reduced FPGA resource usage. A large number of modeled frequencies are possible.

In addition to processing requirements, the implementation complexity may also be increased by timing requirements. For example, the fixed-length numerical integration of the coherent demodulator results in sinc(·) frequency responses the zeros of which are related to *F*_s_/*f_i_*. This results in a limited number of possible high-bandwidth configurations. At low bandwidths, there is much more flexibility in achieving a desired bandwidth as the (*N* + 1)-FIR filter is longer. Here, the group delay (*N*/2) introduced should be considered with respect to the phase margin of the *z*-axis feedback loop.

### Off-mode rejection

Each multifrequency demodulator was assessed by applying a single-tone sine sweep of the carrier frequency ω*_i_* on an input signal described by Equation 1. For each demodulator, an amplitude magnitude frequency response of all three channels was recorded as the input carrier frequency was swept from DC to 750 kHz with a constant amplitude *A_i_*. The three channels were configured to model carrier frequencies of 50, 150, and 300 kHz. In this experiment, the noise floor is dictated by a residual DC offset, which is present due to the finite resolution of the DAC. However, as each demodulation technique was analyzed by using the same hardware, the relative OMR differences are a good indication of each methods performance.

**Figure 8:**
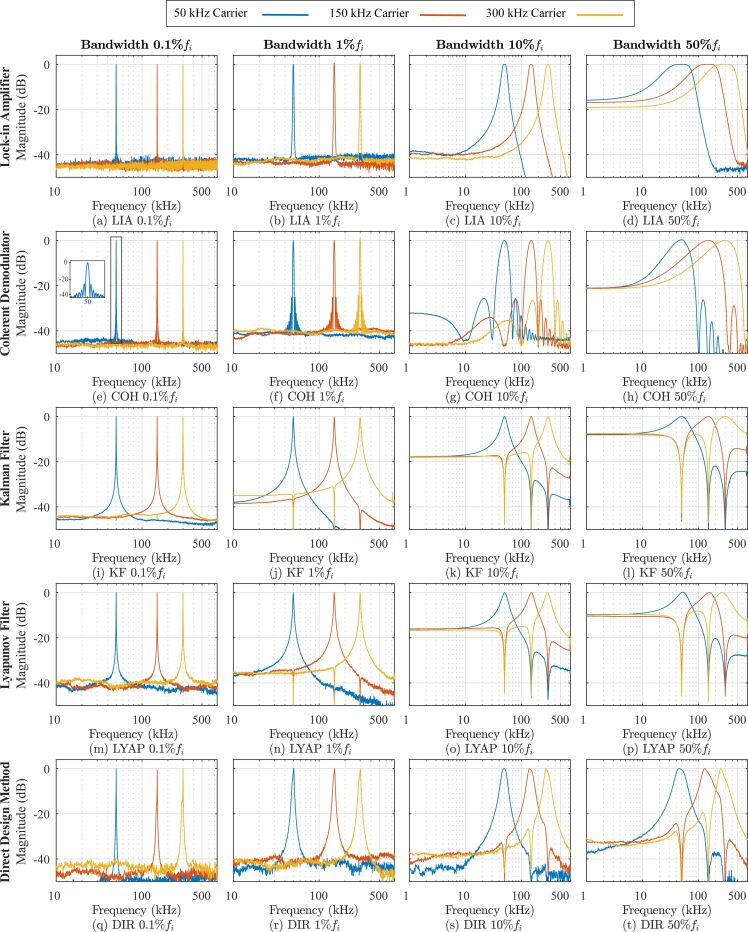
Experimental off-mode rejection results. Here each multifrequency demodulator is on a single row and the tracking bandwidths are adjusted per column with settings of 0.1%*f_i_*, 1%*f_i_*, 10%*f_i_* and 50%*f_i_*. For each system the three modeled carrier frequencies are *f*_1_ = 50kHz (blue), *f*_2_ = 150 kHz (red) and *f*_3_ = 300 kHz (yellow).

The open-loop methods have the benefit of being easily configured to a desired filter order. For this experiment they are of 4th order, which for low-bandwidth settings in Figure 8 creates very steep roll-offs for the lock-in amplifier (Figure 8a,b) and coherent demodulator (Figure 8e,f). When compared to the fixed 1st-order Kalman filter (Figure 8i,j) and Lyapunov filter (Figure 8m,n), the open-loop methods achieve stronger attenuation around the modeled carrier frequency.

The difference between the lock-in amplifier and coherent demodulator is the method used to employ the low-pass filter for suppressing mixing products. In this experiment, the lock-in amplifier utilizes a Butterworth filter, which generates a maximally flat frequency response around the modeled carrier frequency. Conversely, the coherent demodulator employs fixed-length numerical integration resulting in a sinc(·) envelope in its frequency response [28]. This leads to strong OMR at regular intervals at sinc(·) zero locations. However, there is less rejection in-between zeros compared to the Butterworth response.

In contrast to the open-loop methods, the Kalman and Lyapunov filters operate in a closed-loop configuration resulting in state cross-coupling during feedback. As seen in Figure 8 for the Kalman filter (Figure 8i–l) and the Lyapunov filter (Figure 8m–p), this leads to each channel zeroing frequency components corresponding to the other modeled channels. The direct-design method alleviates the fixed 1st-order frequency response of the Kalman and Lyapunov filters. In Figure 8, the direct-design method (Figure 8q–t) performance is shown when configured to a 2nd-order Butterworth filter. The higher filter order results in greater suppression of broadband noise and other frequency components around the modeled carrier frequency when compared to the other closed-loop methods.

Table 2 examines the channel-to-channel OMR performance of each multifrequency demodulator for the 300 kHz channel. It is clear that the open-loop demodulators have a significant performance decrease as the tracking bandwidth increases. The poor OMR is caused by insufficient roll-off of each frequency response with respect to the other modeled frequencies *f*_1_ and *f*_2_. This occurs despite the coherent demodulator rejecting its own 2*f_i_* mixing products. In contrast, the closed-loop Kalman filter, Lyapunov filter and direct-design methods benefit from cross-coupling zeros across all bandwidths allowing them to maintain a strong OMR. The ability to precisely resolve the zeros is limited by the DAC resolution. However, the performance distinction between open-loop and closed-loop methods is clear.

**Table 2:** Channel-to-channel off-mode rejection for the *f*_3_ = 300 kHz channel.

method	Bandwidth 0.1%*f_i_*		Bandwidth 1%*f_i_*		Bandwidth 10%*f_i_*		Bandwidth 50%*f_i_*	
	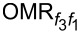	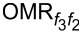	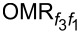	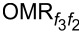	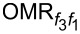	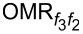	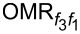	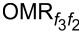
	(dB)	(dB)	(dB)	(dB)	(dB)	(dB)	(dB)	(dB)

lock-in amplifier	−43.8	−44.0	−42.2	−42.8	−39.5	−29.0	−14.2	−3.1
coherent demodulator	−45.6	−46.3	−40.5	−40.0	−36.4	−27.8	−14.3	−4.6
Kalman filter	−44.0	−44.1	−53.0	−52.1	−47.8	−49.7	−42.6	−47.0
Lyapunov filter	−41.7	−40.5	−52.8	−53.1	−44.8	−46.2	−46.1	−47.4
direct-design method	−41.6	−46.7	−42.3	−43.5	−47.2	−46.0	−51.7	−52.3

### Time-domain estimation analysis

**Figure 9:**
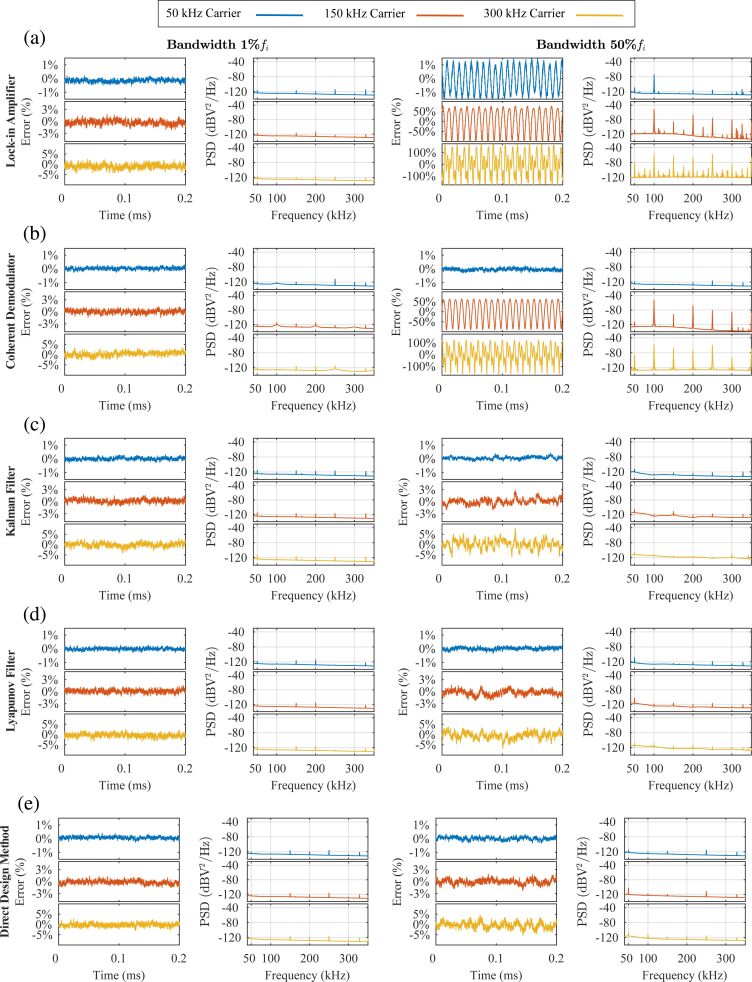
Experimental amplitude estimation error and power spectral density of amplitude estimation for the (a) lock-in amplifier, (b) coherent demodulator, (c) Kalman filter, (d) Lyapunov filter and (e) direct-design method for low (1%*f_i_*) and high (50%*f_i_*) tracking bandwidths. The input signal is described by Equation 2, where *A*_1_ = 500 mV, *A*_2_ = 100 mV, *A*_3_ = 50 mV and *f*_1_ = 50 kHz, *f*_2_ = 150 kHz and *f*_3_ = 300 kHz.

Amplitude estimation performance of the three-channel multifrequency demodulators was investigated when a three-tone sinewave was applied as an input signal described by Equation 2. Here, *A*_1_ = 500 mV, *A*_2_ = 100 mV, *A*_3_ = 50 mV, *f*_1_ = 50 kHz, *f*_2_ = 150 kHz, and *f*_3_ = 300 kHz. Figure 9 shows the amplitude estimation error (
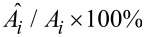
) in the time-domain and amplitude estimate power spectral density (PSD) for both low (1%*f_i_*) and high (50%*f_i_*) tracking bandwidth settings.

When the three-tone sinewave is applied, the performance of each demodulator at low bandwidths is shown to be similar. Each channel is able to estimate the amplitude of its modeled frequency, while strongly attenuating the other frequency components present in the input signal.

At high bandwidths, closed-loop demodulators benefit from cross-coupling zeros at the modeled frequencies. Compared to the open-loop methods, this results in significantly less estimation error as seen in Figure 9. In Figure Figure 9a, the lock-in amplifier 50 kHz estimate contains mixing products at 2*f_i_* = 100 kHz in the time-domain, shown as distinct peaks in the PSD. In contrast, the coherent demodulator in Figure 9b strongly attenuates the mixing products. The performance difference is due to the Butterworth filter not sufficiently attenuating the mixing products, while the sinc(·) envelope contains a zero at 2*f_i_*. However, both open-loop methods poorly estimate the 150 kHz and 300 kHz input signals at high tracking bandwidths due to weak OMR. The PSD reveals that the large estimation errors consist of intermodulation products, which arise from the input multiplying stage.

### AFM imaging

The lock-in amplifier and Lyapunov filter were compared through an MF-AFM imaging experiment where they estimate a signal in the presence of undesirable frequency components. These demodulators were chosen as they are the most simple methods to implement in their respective configurations. This experiment further investigated open-loop and closed-loop demodulator performance at low and high tracking bandwidths.

When compared to bimodal AFM, higher-harmonic AFM [15,60] has inherently greater demodulation challenges. Strong OMR is required as higher harmonics are separated by *nf*_0_, much closer than the approx. 6*f*_0_ second eigenmode spacing [61]. In addition, harmonic content from tip–sample interactions scales with approx. 1/*n*^2^[13]. Therefore, the signals of interest are detected in the presence of a much larger fundamental resonance frequency, emphasizing the need for strong noise sensitivity from the demodulator.

Higher-harmonic AFM imaging was performed using an NT-MDT NTEGRA AFM on the second harmonic amplitude. The chosen cantilever (Budget Sensor TAP190G) has a fundamental resonance frequency of 156.75 kHz. The sample is a blend of polystyrene (PS) and polyolefin elastomer (LDPE) available from Bruker (PS-LDPE-12M). Due to the different elastic moduli of the PS and LPDE regions, the sample is used for evaluating imaging methods that are sensitive to elasticity.

Higher-harmonic amplitude images were obtained by the lock-in amplifier and Lyapunov filter on the second harmonic. Each demodulator was configured to track 313.50 kHz. In addition, the Lyapunov filter contained a channel modeling the fundamental resonance frequency. Although the cantilever is actively driven at its fundamental resonance frequency, during imaging its deflection signal contains additional frequency components. These include higher harmonics and intermodulation products excited by non-linear tip–sample forces during contact.

**Figure 10:**
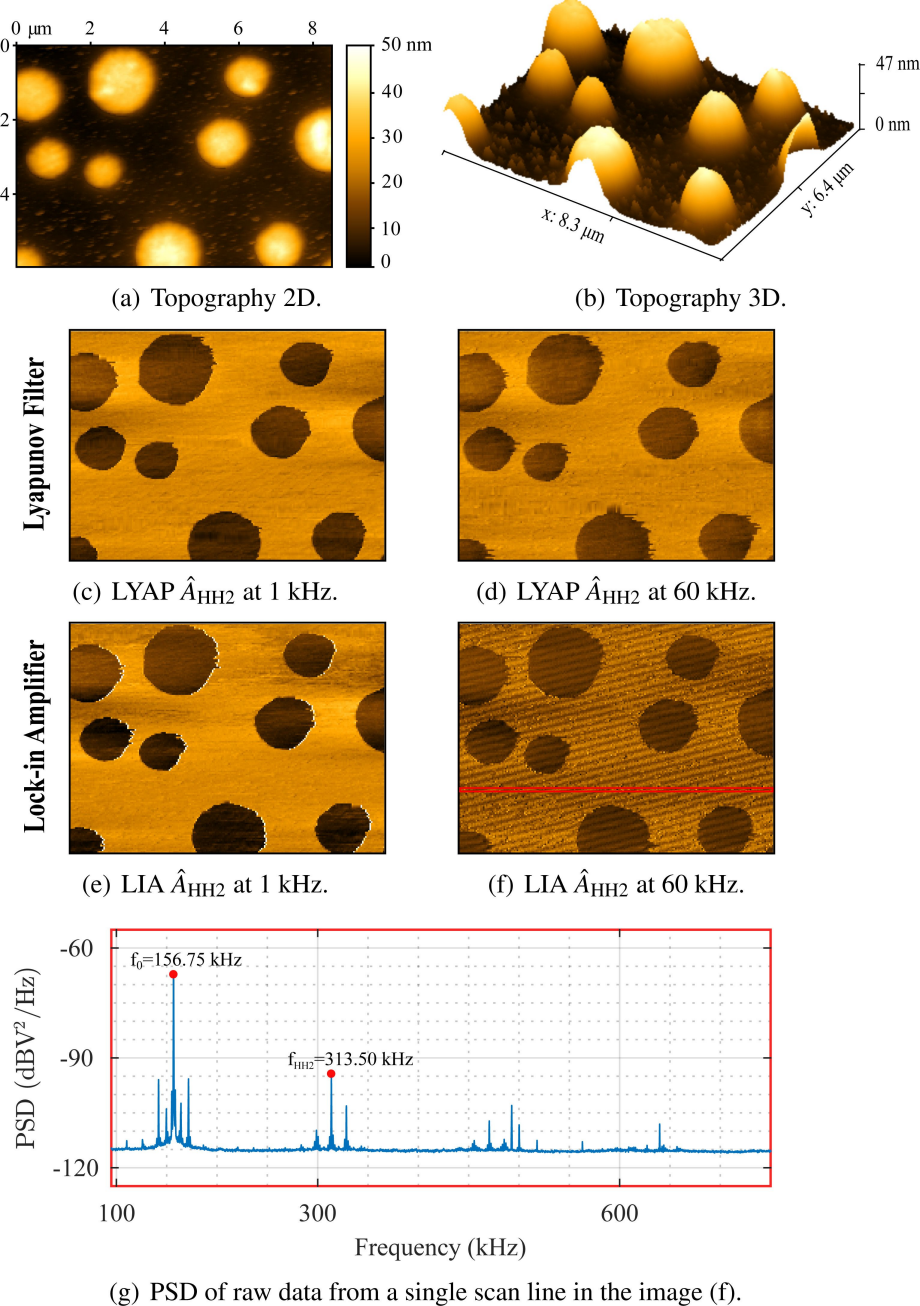
Higher-harmonic amplitude AFM imaging performed with the fundamental mode of a TAP190G cantilever on a PS/LPDE polymer blend. Images shown are the (a,b) topography in nanometers at 3 kHz, with parallel second-harmonic amplitude estimates from the (c,d) lock-in amplifier and (e,f) Lyapunov filter at tracking bandwidths of 1 kHz and 60 kHz. The PSD (g) is shown for the raw data of a single scan line from the image in (f).

Second-harmonic amplitude images captured by both demodulators at low (1 kHz) and high (60 kHz) tracking bandwidths are shown in Figure 10. At low bandwidths, the lock-in amplifier (Figure 10e) and the Lyapunov filter (Figure 10c) perform comparably as demonstrated in a previous work [36]. However, at high bandwidths the lock-in amplifier image has large artifacts when compared to the Lyapunov filter. This is due to the different OMR achieved by each system with respect to the fundamental resonance frequency. Through channel cross-coupling, the Lyapunov filter is guaranteed to contain a zero at the desired location of 156.75 kHz.

In contrast, the lock-in amplifier insufficiently attenuates the fundamental resonance frequency. The PSD of the raw data from a single scan line (Figure 10g), taken from the image in Figure 10f, reveals that the estimate contains large intermodulation products. These signal components are aliased due to the low AFM sampling frequency (*F*_s_ = 256 Hz), resulting in the low-frequency artifacts seen in Figure 10f.

## Conclusion

This article compares the performance of traditional and recently proposed demodulators for MF-AFM. These include conventional open-loop methods such as the lock-in amplifier and coherent demodulator, and closed-loop methods such as the Kalman filter, the Lyapunov filter and the direct-design method. The sensitivity of each demodulator to unwanted frequency components was assessed for low and high tracking bandwidths. Additionally, higher-harmonic AFM imaging was conducted for both low and high tracking bandwidths to further compare demodulator performance.

Open-loop demodulation schemes attenuate the high-frequency mixing component at 2*f_i_* by employing a low-pass filter. The lock-in amplifier provides flexibility to implement a desired filter response and order. Conversely, the coherent demodulator contains a sinc(·) envelope as it performs numerical integration over a fixed-length time window. Both demodulators excel at low bandwidths due to steep roll-offs, while having poor OMR at high tracking bandwidths. Although the lock-in amplifier implementation is simpler, the coherent demodulator sinc(·) lobes are advantageous for higher harmonic and intermodulation AFM.

The closed-loop Kalman filter, Lyapunov filter and direct-design method employ internal feedback of the estimated states to reject the mixing products. This allows them to maximize the tracking bandwidth without introducing additional noise in the amplitude estimate [28]. An added benefit of this approach is cross-coupling zeros occurring at modeled frequencies, which was demonstrated to reduce estimation artifacts. The direct-design method alleviates the limited 1st-order response of the Kalman and Lyapunov filters. When configured to a 2nd-order Butterworth response, it achieved an increased roll-off which increases broadband noise suppression while still maintaining strong OMR performance.

Table 3 is provided as a reference of MF-AFM application characteristics and required demodulator properties. A recommendation for which demodulator is most suited to three major MF-AFM applications is given as follows:

**Intermodulation AFM**: This MF-AFM application tracks a large number of closely spaced intermodulation products [16]. As each signal of interest has a frequency separation of the order of 100 Hz, a very low bandwidth and a very strong OMR are essential. The requirement to track up to 40+ signals is most suited to a computationally inexpensive open-loop method. The coherent demodulator is recommended for intermodulation AFM, since the sinc(·) response of each channel can be configured to zero other intermodulation products [58].

**Higher-harmonic AFM**: This MF-AFM application tracks integer multiples of the cantilever fundamental resonance frequency, resulting in frequency spacing of the order of the fundamental resonance frequency, which ranges between 100 and 300 kHz. Since each harmonic is in the presence of a much larger fundamental resonance frequency, a low tracking bandwidth and a strong OMR is required. The open-loop lock-in amplifier and coherent demodulator are recommended at low tracking bandwidths. While the closed-loop Lyapunov filter and direct-design method are recommended if a higher tracking bandwidth is desired. This is because the closed-loop methods have the added benefit of zeroing the large fundamental resonance frequency and other harmonics.

**Higher-mode AFM**: This MF-AFM application tracks the fundamental resonance frequency and higher resonance modes. Frequency content of interest is typically separated by 500 kHz or more, depending on the cantilever geometry. This provides flexibility to the user to operate at either a low or high tracking bandwidth. At low tracking bandwidths, the lock-in amplifier is recommended, as it is of lower complexity than the coherent demodulator, which offers little benefit for widely spaced signals. At high tracking bandwidths, a closed-loop method is recommended as they achieve single-cycle convergence (*f*_−3dB_ ≈ *f*_i_) with optimal noise performance. Also, each channel has the added benefit of zeroing other resonant modes. The Lyapunov filter and direct-design method are preferred over the Kalman filter, as they are significantly easier to implement.

**Table 3:** Summary of MF-AFM applications and required demodulator properties. Note: *f*_0_ is the fundamental resonance frequency and *f_i_* is the demodulated frequency.

MF-AFM application	application characteristics		demodulator properties	
	frequency spacing	number of channels	tracking bandwidth	off-mode rejection

intermodulation	very small	40+	very low	very important
	(*f_i_*/*f*_0_ ≪ 1)		(≪1%*f*_0_)	
higher harmonic	medium	10+	low	important
	(*f_i_*/*f*_0_ ≈ 1)		(1%*f*_0_)	
higher mode	large	1–5	user choice	less important
	(*f_i_*/*f*_0_ ≫ 1)		(≥1%*f*_0_)	
